# Diatom Frustule Array for Flow-Through Enhancement of Fluorescent Signal in a Microfluidic Chip

**DOI:** 10.3390/mi12091017

**Published:** 2021-08-26

**Authors:** Zhenhu Wang, De Gong, Jun Cai

**Affiliations:** School of Mechanical Engineering and Automation, Beihang University, Beijing 100191, China; zhwangstayhungry@buaa.edu.cn (Z.W.); gongde@buaa.edu.cn (D.G.)

**Keywords:** diatom frustules, array, flow-through, microfluidic chip, POCT, biosensing

## Abstract

Diatom frustules are a type of natural biomaterials that feature regular shape and intricate hierarchical micro/nano structures. They have shown excellent performance in biosensing, yet few studies have been performed on flow-through detection. In this study, diatom frustules were patterned into step-through holes and bonded with silicon substrate to form an open-ended filtration array. Then they were fixed into a microfluidic chip with a smartphone-based POCT. Human IgG and FITC-labeled goat–anti-human IgG were adopted to investigate the adsorption enhancement when analyte flowed through diatom frustules. The results indicated up to 16-fold enhancement of fluorescent signal sensitivity for the flow-through mode compared with flow-over mode, at a low concentration of 10.0 μg/mL. Moreover, the maximum flow rate reached 2.0 μL/s, which resulted in a significant decrease in the testing time in POCT. The adsorption simulation results of diatom array embedded in the microchannel shows good agreement with experimental results, which further proves the filtration enrichment effect of the diatom array. The methods put forward in this study may open a new window for the application of diatom frustules in biosensing platforms.

## 1. Introduction

Diatom frustules, a type of nanostructured biomaterials synthesized by diatoms, are famous for their hierarchical regular pores structure composed of amorphous silica. Their delicate structures and composition have drawn considerable attention in recent decades, and are verified to endow diatom frustules with excellent properties, such as outstanding mechanical strength [1], high specific surface ratio [2,3], biocompatibility [4,5], and transparency. Based on these excellent properties, diatom frustules have been widely used in a variety of technological applications where they are regarded as a promising alternative to synthetic materials, including drug delivery [6], immunoassay detection [7], battery electrodes [8], chemical modification [9], and filtration [10]. Among frequently used diatom frustules, a typical example is *Coscinodiscus* sp. (*C.* sp.) frustules. They are petri-dish shaped with three layers of pores, of which the diameters range from 40 nm to 1 μm. Their frustule valves can be regarded as “micro-sieves”, when similar artificial porous membranes are often used for the filtration and separation process. Compared with synthetic porous membranes, diatoms reproduce at an exponential rate and therefore may offer cheap nanoporous silica. In addition, diatoms live in a complex environment where they are exposed to nutrient molecules, deleterious bacteria, and viruses. Their frustules have evolved specific structures and functions that help them survive in a marine environment [11]. From that point of view, diatom frustules have an optimized filtration and separation membrane, which shows great potential in microfluidic systems.

Although considerable efforts have gone into the use of diatom frustules, to the best of our knowledge, diatom frustules have been mainly used as a kind of mesoporous material in most previous research [12,13,14,15,16,17,18]. A few studies have considered their various shapes/structures and ordered distribution [19]. There remain some challenges to integrate diatom frustules into microfluidic chips as well as flow-through sensing, e.g., a lack of arrangement techniques to pattern diatom frustules into array forms precisely and conveniently, as pointed out by Dusan Losic et al. [11], who fixed an individual diatom at the end of a capillary tube to explore selective membrane separation. Their work could be regarded as the prototype and first attempt to combine diatom frustules with microfluidics. Later, Kong et al. [20] presented a microfluidic chip integrated with diatomite for sensing illicit drugs at an ultra-high detection sensitivity down to the 1-ppb level, which achieved an improvement factor of 1000 times that of a normal chromatography plate device. When reaching a high sensitivity, they used massive out-of-order frustules as investigation objects. Great effort has been made to realize uniform distribution of diatom frustules from a state of disorder, including monolayer assembly [21,22,23] and regular array [24,25,26]. The latter is more challenging, but shows promise in lab-on-chip detection.

In this study, the diatom frustule array was obtained by a template-assisted method, followed by integration into a microfluidic chip to realize the low through-sensing of antigen–antibody tests in an active filtration manner. We also built smartphone-based POCT devices to capture and process a fluorescence signal. The silicon template with step-through holes was etched using a conventional MEMS technique. To obtain different orientations of the diatom frustules array, a micromanipulator was used to arrange frustules in the holes. A spontaneous coating method was put forward to bond frustules with silicon substrate. Finally, the substrate with frustule array was modified and fixed into a PMMA microfluidic chip. Frustules with different orientation showed nearly equal effect in filtrable adsorption for flow-through detection. We also fabricated the flow-over sensing chip as a contrast. The results indicated a maximum of 16-fold improvement in fluorescence signal sensitivity, at a high flow rate of up to 2.0 μL/s and low concentration of 10.0 μg/mL. The methods put forward in this study may promote the application of diatom frustules in biosensing platforms.

## 2. Materials and Methods

### 2.1. Materials and Characterization

The cultivated *Coscinodiscus* sp. diatoms (CCMP 1583) were purchased from the National Center for Marine Algae and Microbiota (NCMA) at the Bigelow Laboratory for Ocean Sciences, East Boothbay, ME, USA. The methods to obtain clean frustules can be found in our previous research [27]. Phosphate-buffered solution (PBS, 10×, pH 7.2–7.4), Human IgG and FITC-goat–anti-human IgG were purchased from Solarbio^®^ Life Science, Beijing, China. 3-aminopropyltriethoxysilane (APS) was purchased from Cool Chemistry Inc., Beijing, China. Methylbenzene and ethyl alcohol was purchased from Modern Oriental Fine Chemistry^®^, Beijing, China. EVA hot-melt glue was purchased from Deli Group^®^, Ningbo, China. Lens pair was purchased from Aladdin^®^, Beijing, China. Interference filters and long-pass filters were purchase from Zolix^®^, Beijing, China. LEDs (blue, 3 W) and cooling holders were purchased from Shenzhen Taiyi Photoelectric Co., Ltd, Shenzhen, China. PMMA substrate (0.5 mm) was purchased from Taobao, Beijing, China.

The morphology of diatom frustules and micro-step-through holes were observed by field-emission scanning electron microscopy (FE-SEM) (Hitachi SU-8010, Hitachi, Tokyo, Japan). A micromanipulator (Heidstar Co., Ltd., Xiamen, China) was adopted to pattern frustules. A plasma cleaning machine (YZD08-2C, SEOT(Beijing) Technology Co., Ltd., Beijing, China) was used to reduce the contact angle of Si substrate. An electric iron with constant temperature (SS-936A, Wenzhou Hanbang Electronic Co., Ltd., Wenzhou, China) was used to heat the hot-melt glue. A laser cutter (E1309M, Zhengtian Hengye CNC technology Co., Ltd., Beijing, China) was used to cut the PMMA substrate to be combined into microfluidic chips. A syringe pump (PHD Ultra, Harvard Apparatus, Holliston, MA, USA) was used to inject solution to the microfluidic chip.

### 2.2. Fabrication of Si Substrate with Step-Through Holes

Conventional MEMS techniques were adopted to etch the micro-step-through holes, as shown in Figure S1 in Supplementary Materials. First, a local back cavity was fabricated using the wet etching technique to reduce the thickness of the Si wafer from 400 μm to 50 μm. A nano-layer of SiO_2_/Si_3_N_4_ was grown on the surface of the wafer, which will be used as the mask to etch the bigger holes. Next, using a photoresist as mask, the SiO_2_/Si_3_N_4_ nano-layer was etched while the Si wafer remained unetched. Then, a photoresist layer was re-coated on the wafer and used as the mask to etch smaller holes 45 μm in depth. Finally, the photoresist was removed, and the patterned SiO_2_/Si_3_N_4_ nano-layer was used as the mask to etch bigger holes 15 μm depth. In this process, the smaller holes would also be etched through.

### 2.3. Coating Hot-Melt Glue in Micro-Holes under Capillary Force and Bonding Frustules

EVA hot-melt glue is insoluble in methylbenzene at room temperature. First, methylbenzene was added into a 50 mL centrifugal tube and heated to 95 °C in a water bath. Then, the EVA powder was added to the methylbenzene at 95 °C for 30 min. Finally, the tube was taken from the water bath and cooled to the room temperature. The concentration of EVA hot-melt glue solution is 0.05 g/mL. This procedure was finished in a fume hood. The detailed process of “spontaneously coating under capillary force” is demonstrated in Figures S2–S4 in Supplementary Materials.

### 2.4. Patterning Frustules by a Micromanipulator

A micromanipulator platform was built to manipulate diatom frustules into an array pattern, and the detailed operating procedures are demonstrated in Figure S5 in Supplementary Materials. Briefly, a single frustule was attached to a capillary glass microneedle under electrostatic force and transferred to the position right above a micro-step-through hole. Then, negative pressure was exerted from the back of the Si substrate to attract the frustule to detach from the microneedle and fall into the micro-hole. By choosing frustules with different orientations in the first step, a frustule array with different orientations could be obtained.

### 2.5. Modification of Diatom Frustules and Antigen–Antibody Test

A 10 mL 2% solution of APS in ethyl alcohol and 50 mL 5.0 μg/mL solution of purified primary human IgG in PBS was prepared. A total of 10 mL FITC-labeled goat–anti-human IgG of 10.0 μg/mL concentration was prepared in a dark room.

The Si substrate with the frustule array was immersed into APS solution for 90 s for surface modification, then washed with ethyl alcohol for 1 min. After drying at room temperature, the substrate was immersed into human IgG solution at 38 °C for 12 h for incubation [26]. Finally, the substrate was washed with deionized water for 1 min and dried naturally, then fixed into a PMMA microfluidic chip.

Different volumes (0 μL to 200 μL) of FITC-labeled goat–anti-human IgG were injected to the microchannel and driven to flow-through frustules under a negative pressure of −64 kPa, which was exerted by a vacuum pump. Then 50 μL PBS solution was injected into the microchannel to wash the residual antigen and antibody solution.

### 2.6. PMMA Microfluidic Chip Fabrication and Bonding

AutoCAD was used for the design, and a laser cutter was used to cut PMMA into pieces, each of which had different structures and played different roles. UV curing adhesive was used to bond the four pieces of PMMA and Si substrate, as shown in Figure S6 in Supplementary Materials.

## 3. Results and Discussion

### 3.1. Diatom Frustule Morphology and Array

**Figure 1:**
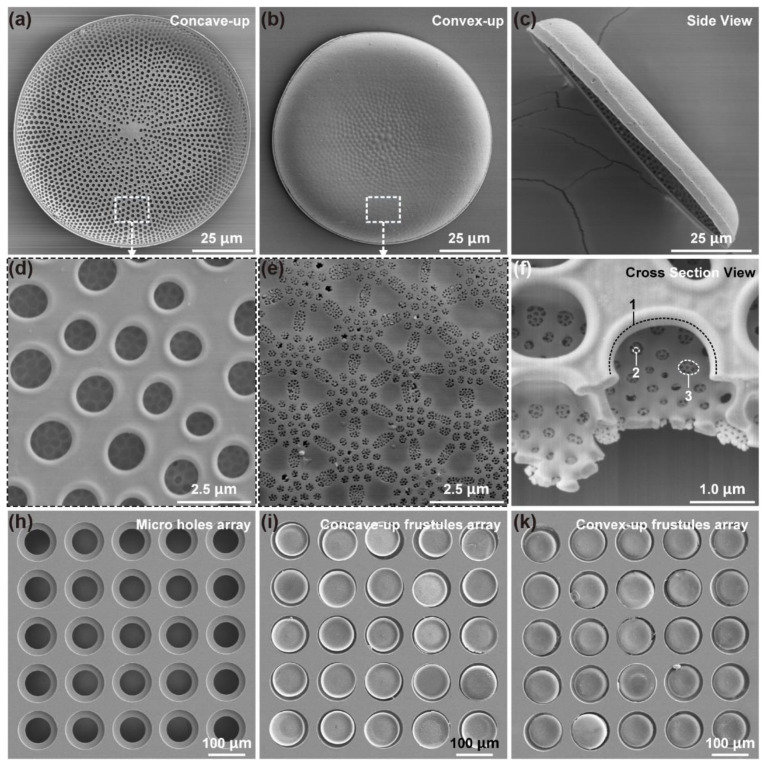
The morphology and array of *C.* sp. frustules. (**a**) The concave side with evenly distributed ~1 μm pores; (**b**) The convex side with uniformly distributed ~200 nm and ~40 nm pores; (**c**) The side view of a diatom frustule indicating the height is about 10 μm; (**d**) The detailed morphology of the concave side; (**e**) The detailed morphology of the convex side; (**f**) The cross section of a diatom frustule, where three layers of pores can be clearly observed. (**h**) SEM images of the step holes; the diameters are 115 μm and 75 μm, and center distance is 138 μm. The depth of the bigger hole is around 10 μm. (**i**) Concave-up frustules array and (**k**) convex-up frustules array, in which each frustule is bonded with the Si substrate by hot-melt glue.

The morphology of *C.* sp. diatom frustule is shown in Figure 1a–f. The average diameter is about 90~100 μm, and the average thickness is about 1 μm, as shown in Figure 1a,b. The average height is about 10 μm, as shown in Figure 1c. The frustules could be regarded as bowl-shaped micro-sieves, where three layers of pores are regularly synthesized by diatom cells from the concave surface to the convex surface, with diameters of ~1 μm, 200 nm, and 40 nm, respectively, as shown in Figure 1f, label 1–3 (usually called foramen, cribrum, and cribellum, respectively). The micro/nano-pores are distributed uniformly, as shown in Figure 1d,e. Between the micro-pores layer and nano-pores layer, there is a hexagonal chamber.

To obtain the frustule array, conventional MEMS technique was adopted to fabricate micro-step-through holes on silicon substrate, as shown in Figure 1h, which was used as the limit template to fix the diatom frustules. The detailed etching process is demonstrated in Figure S1 in Supplementary Materials. The diameters and center distance of micro-holes could be designed as required. In this study, the diameters of bigger and smaller holes were 115 μm and 75 μm, respectively. The center distance was 138 μm, which was 1.2 times the diameter of the bigger holes. The depth of the bigger holes was around 13 μm, which was bigger than the height of the diatom frustules to provide protection to the micro-nano structure integrity.

The 5 × 5 diatom frustule array with different orientations were arranged using a micromanipulator (Figure S4 in Supplementary Materials), and bonded with the silicon substrate by hot-melt glue, as shown in Figure 1i,k. Diatom frustules were precisely positioned into the holes, so that fluid could be forced to pass though the hierarchical pore structure. The diatom frustules can be regarded as “micro-sieve” to filter and adsorb target molecules or particles in fluid. However, the frustules are unsymmetrical in pore distribution order along the normal direction, which would lead to a difference in flow field. Therefore, frustule arrays with different orientations were prepared to test the adsorption performance.

Additionally, we proposed a new method to immobilize frustules in the holes reliably. Frustules are composed of amorphous silica (SiO_2_×H_2_O), which do not react with silicon directly. Although there have been many studies that put forward bonding methods to immobilize frustules, we believe that most of them are unsuitable for this study after carefully consideration. For example, electrostatic force is too weak to fix frustules [24]; HF bonding needs to be realized under a certain pressure and HF would destroy the nano structure of frustules [28]. Hot-melt glue is an ideal candidate for use as a middle layer to bond frustules with a Si substrate, which was evaluated in our previous research [26]. One challenge is to restrict the hot-melt glue to micro-holes to keep the surface of the Si substrate clean. We demonstrated an innovative method called “spontaneously coating under capillary force”. The principle and detailed process are shown in Figure S2 in Supplementary Materials. Using this method, the frustules were immobilized in the holes tightly, and did not detach from Si substrate.

### 3.2. Diatom Modified Process and POCT Devices

**Figure 2:**
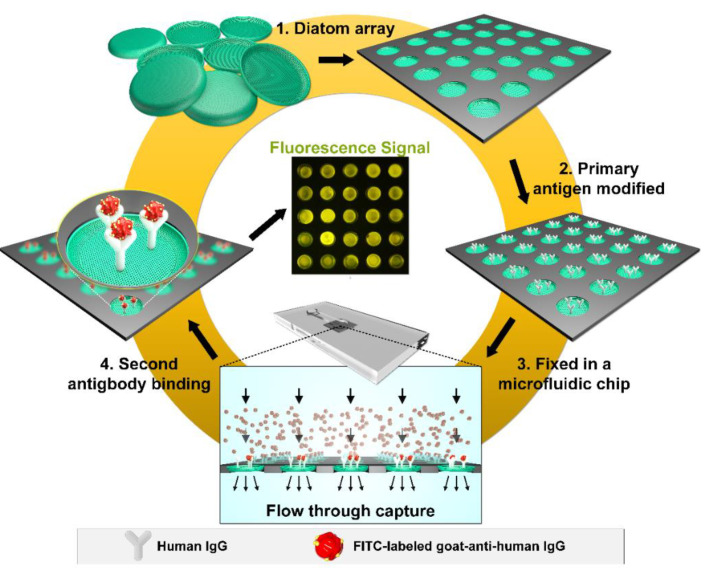
Schematic illustrations of the frustule array modification and detection.

A fluorescent antibody test was used to investigate the adsorption performance of the frustule array under flow-through condition, as shown in Figure 2. The outstanding adsorption performance of the diatom frustules have been verified by numerous excellent studies [29,30,31]. Photonic crystal effects of diatom frustules are also beneficial to the enhancement of the optical signal [32]. When used in fluorescent sensing, the porous structure of the frustules could improve the stability of incorporated fluorophores by protecting them from photo-bleaching [33]. The frustule surface was first modified by primary antigen human IgG to yield a selective surface. The substrate with the frustule array was then fixed in a microfluidic chip. The goat–anti-human IgG would bind to the human IgG that was evenly distributed across the surface of the frustules. Since the solution flows through the frustules, the flow rate was set to be 2.0 μL/s. Therefore, the reaction time was reduced effectively to less than 5 min in this investigation.

**Figure 3:**
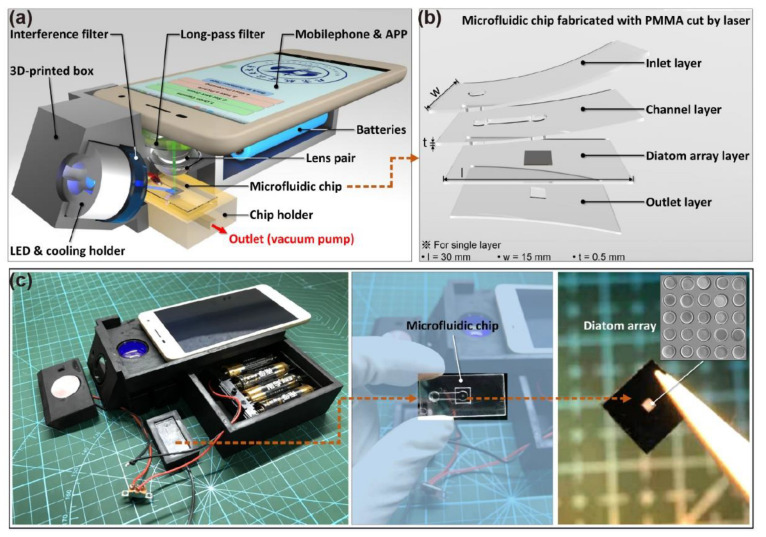
Schematic illustration of (**a**) POCT devices and (**b**) microfluidic chip. (**c**) A picture of the POCT devices.

The schematic illustration of POCT devices based on a smartphone is shown in Figure 3a. The microfluidic chip with the frustule array was placed in a chip holder which was connected to a vacuum to drive the solution to flow in the channel. The microfluidic chip consisted of four pieces of PMMA. The microchannel is 3.0 mm wide and cut by a laser cutting machine. UV curing adhesive was used to fix and seal the Si substrate in the chip and to bond four layers of PMMA to form a microfluidic chip, as shown in Figure 3b. Cutting PMMA chips by laser is now a popular method to fabricate microfluidic chips on account of its high efficiency. However, the height of the microchannel was 500 μm in this study, which was larger than that of PDMS chips, since the minimum thickness of the PMMA we could purchase was 500 μm. Moreover, the width of the microchannel was decided by the laser types. In this study, a carbon dioxide laser was used to cut PMMA, thus the minimum width of the microchannel was 3.0 mm. Therefore, this chip was more suitable for high-throughput detection.

A picture of the POCT devices is shown in Figure 3c. These POCT devices were designed to be applied in FITC detection. By selecting different interference filters and long-pass filters, it could also recognize other fluorescent molecules. However, most smartphones on the market use a CMOS sensor in the camera, which is usually more sensitive to green than red and yellow. Therefore, FITC was selected as the fluorescent molecule with emission wavelength 518 nm. Moreover, when compared with the commonly used fluorescence microscope, whose mercury lamps usually reach 1000 W, the power of the single LED in this POCT was 3W as the excitation light source, which was quite low, and the fluorescence signal was weak. Although the camera of smartphone is sophisticated and precise, it still performed unsatisfactorily in capturing light in dark environments, especially with a fluorescent signal. The large specific surface area of the nanostructured frustules is known to increase antigen capture [34], which could enhance weak fluorescence. Moreover, the magnification of the lens pair is 10×, while the maximum optical zoom of the smartphone is also 10×, which could realize the observation of diatom frustules.

### 3.3. User Interface and Working Principle

**Figure 4:**
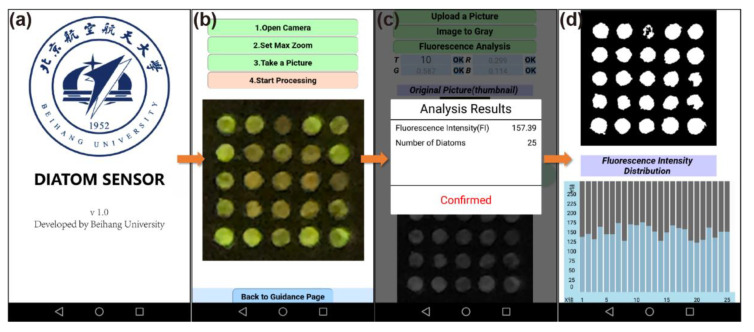
Pictures of the app interface. (**a**) Start-up screen; (**b**) Fluorescence photo capture interface; (**c**) Photo-processing interface and result output; (**d**) Binary map of fluorescence photo and distribution chart of fluorescence intensity.

We developed an Android app called *Diatom Sensor* to process the fluorescence photos on the smartphone in situ and output the average fluorescence intensity of 25 frustules, as well as the fluorescence intensity of each frustule, as shown in Figure 4. The operating procedure is quite simple and user-friendly, which is demonstrated in Figure 4b. The average fluorescence intensity was output directly, as shown in Figure 4c. The white area in the binary map in Figure 4d stands for diatom frustules, and the bar chart shows the distribution of fluorescence intensity.

**Figure 5:**
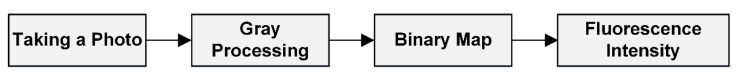
Working principle of the app for fluorescence signal processing.

The principle to obtain fluorescence intensity is shown in Figure 5. Briefly, the original fluorescence photo is first transformed to a gray map based on following equation:

(1) Gray value=0.299×rValue+0.587×gValue+0.114×bValue

where rValue, gValue, and bValue represent the (*R*, *G*, *B*) value of each pixel of the picture. The Gray value is between 0 and 255. Then a threshold is calculated based on the gray map using the OTSU method [35]. If the Gray value of a pixel is bigger than the threshold, this pixel is set to be white; otherwise, it is set to be black. Thus, the frustules area can be recognized. Finally, the fluorescence intensity of each frustule is calculated based on the following equation:

(2) $$\text{Fluorescence Intensity}=\frac{\sum Gray\;vlaue}{Diatom\;Area}\;$$

where ∑Gray vlaue represents the sum of the Gray value of every pixel in the diatom area. Therefore, the final fluorescence intensity obtained by this method lies in [0, 255].

### 3.4. Capture Performance of Flow-Through vs. Flow-Over Test

**Figure 6:**
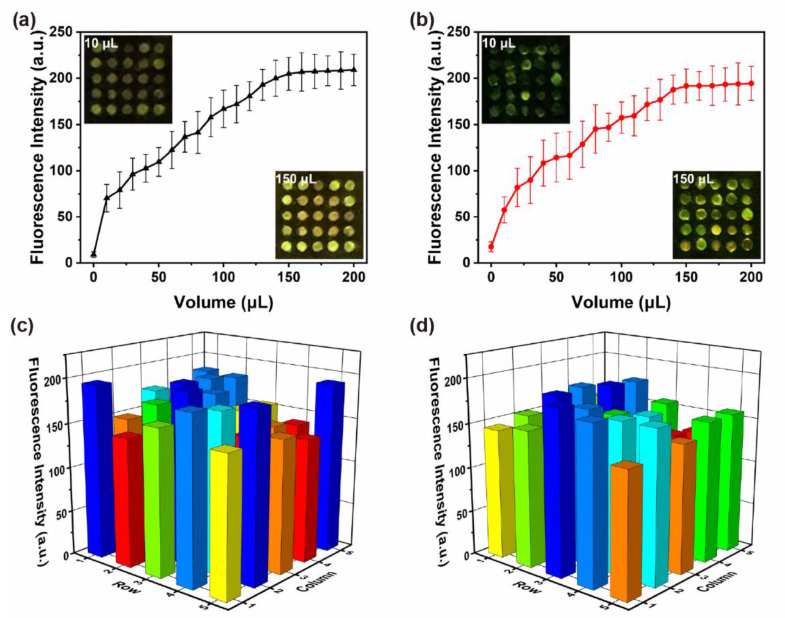
The average fluorescent intensity of (**a**) concave-up frustules and (**b**) convex-up frustules. The fluorescence intensity distribution of 25 frustules of (**c**) concave-up orientation and (**d**) convex-up orientation.

Flow-over and flow-through mode are two frequently used techniques in lab-on-chip sensing [36,37,38]. In flow-over assays, molecular binding mainly depends on the transportation of bulk analyte through diffusion and convection [39]. For those sensors based on micro/nano structures or nanoparticle clusters, especially for nanohole sensing, the flow direction that is parallel to the microchannel may encounter limitations, such as the limited diffuse process of molecules into the interior surface, which may result in poor capture efficiency [40]. The earlier formed depletion zone around the sensor surface would also repel follow-up molecules, thus capture capability would be further affected [39]. To improve the efficiency of analyte delivery and eliminate the depletion zone, a flow-through mode that drives the analyte solution passing through the nanoholes is believed to be a practicable technique. A fluorescent antibody test was utilized to investigate the adsorption performance and enrichment effect of frustules, and the results are shown in Figure 6. The fluorescence intensity increased with the volume increasing from 0 μL to 150 μL, as shown in Figure 6a,b. The average fluorescence intensity of concave-up frustules was slightly larger than that of convex-up frustules, but there was no significant difference. The intensity of 25 frustules at 100 μL is shown in Figure 6c,d. The relative standard deviations (RSD) were 20.18% and 16.96% for concave-up and convex-up frustules, respectively. The fluorescence intensity was relatively uniform along the flow direction. It was rational to take the average fluorescence signal as the output results. Moreover, the results indicated that frustules of both orientations could act as enhanced elements for antibody molecules. They played equivalent roles in the capture of antibody molecules.

**Figure 7:**
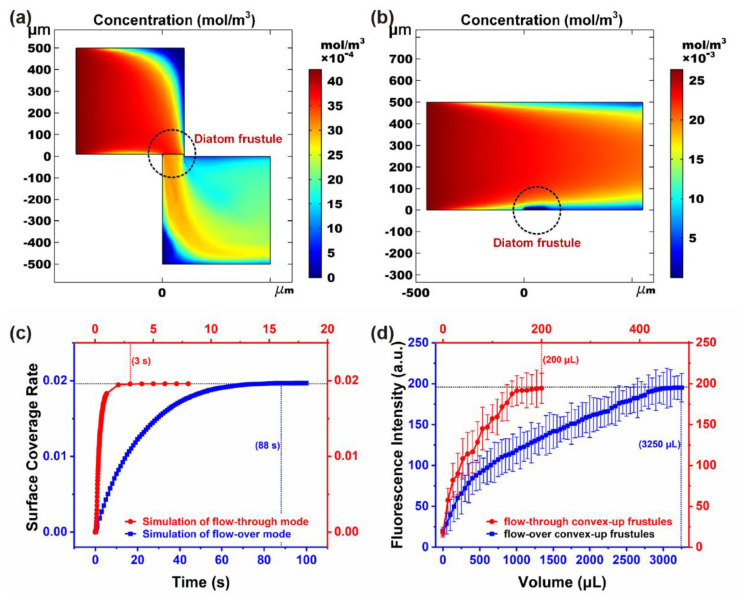
Simulation results of concentration distribution of (**a**) flow-through, (**b**) flow-over detection, and (**c**) surface coverage rate of fluorescence molecules over time. (**d**) Smartphone analysis results of fluorescence intensity of flow-through and flow-over for convex-up frustules.

However, there was a distinct improvement in response time for flow-through tests when compared with flow-over tests, as shown in Figure 7, both in simulation and experimental results. Here, the frustules were in a convex-up orientation, since for concave-up frustules, the pore structure would be filled by hot-melt glue when bonded to a silicon substrate to investigate flow-over performance. When the solution flowed through the diatom frustules, a significant enrichment effect could be observed in Figure 7a. By contrast, for the flow-over test, the height of the microchannel was 50 times that of frustules. When the frustules were fixed on the bottom of the microchannel, where the flow velocity was quite low, the probability of adsorbing molecules for frustules was severely reduced (Figure 7b). Therefore, the response time increased by nearly 29 times for simulation (Figure 7c and Figure S7) and 16 times for experimental results (Figure 7d).

### 3.5. Adsorption Simulation

**Figure 8:**
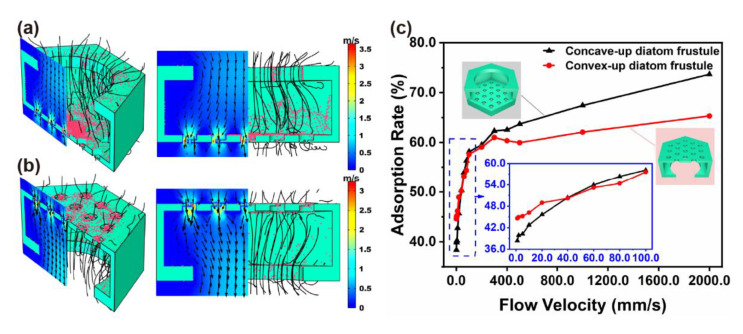
Simulation results of flow field in single-cell and nanoparticle distribution of cell surface for (**a**) concave-up frustules, (**b**) convex-up frustules, and (**c**) adsorption rate via flow velocity.

Flow velocity is a major factor that influences the adsorption performance. However, it is hard to observe the dynamic flow field in the micro/nano-pores of frustules directly. Using the microfluidic module and particle-tracing module of COMSOL Multiphysics, flow field as well as particle adsorption position and rate were analyzed, as shown in Figure 8. The frustules could be regarded as a combination of periodic micro-hexagonal cells, which consisted of one 1 μm pore and dozens of 200 nm pores and 40 nm pores. The model and simulation parameter are shown in Figure S8 in Supplementary Materials. The nano-pores layer played a major role in particle adsorption for both concave-up and convex-up frustules. However, the inner side wall of concave-up cells adsorbed more particles than that of convex-up cells (Figure 8a,b). For concave-up cells, the flow resistance increased from a 1 μm pore to nano-pores, which causes more violent disturbance. Therefore, the contact probability between the particles and side wall improved, which could also be verified by the two-phase flow simulation in Figure S9 in Supplementary Materials. Moreover, the flow rate was 2.0 μL/s, with solution passing through 25 micro-pores of diameter 37.5 μm. Thus, the flow velocity across each frustule could be estimated by the following equation:

(3) $$Flow\;velocity\approx \;\frac{Flow\;rate}{Sectional\;area}=\;\frac{2.0\;\mu L/s}{25\times \pi \times {\left(37.5\;\mu m\right)}^{2}}=56.9\;\mathrm{mm}/s$$

According to simulation results, there was no significant difference in the adsorption rate at a low flow velocity, such as less than 200 mm/s, as shown in Figure 8c, which was consistent with the smartphone processing data.

## 4. Conclusions

In this study, a template with step-through holes was etched to arrange diatom frustules into an array pattern precisely with a micromanipulator, followed by bonding frustules to silicon substrate with hot-melt glue, aimed at improving fluorescence intensity under the enrichment effect of porous biomaterial diatom frustules. A POCT device was developed to test the adsorption performance of the frustule array that was fixed in a PMMA microfluidic chip. Experimental results indicated that a significant improvement in response time of up to 16-fold was realized at a high flow rate, when the solution flowed through the frustules, in comparison with the flow-over status. A simulation was conducted to investigate the adsorption mechanism of the frustules to nanoparticles. Furthermore, as the frustules are composed of silica, they can be applied not only for model antibody goat–anti-human IgG but also other types of immunocomplex formations such as ELISA. The methods put forward in this study can be applied further to diatom frustules in biosensors.

## Supplementary Materials

The following are available online at https://www.mdpi.com/article/10.3390/mi12091017/s1, Figure S1: (a) Procedure to etch micro-step-through holes and (b) SEM image of the holes.; Figure S2: Coating hot-melt glue in micro holes by under capillary force and bonding frustules; Figure S3: Optical images of micro holes with hot-melt glue; Figure S4: SEM images of (a,c) diatom frustules array, that (b,d) bonded to the Si by hot-melt glue; Figure S5: (a) Schematic illustrations of manipulating diatom frustules into array pattern; (b) Pictures of micromanipulator system; Figure S6: Microfluidics chip bonding; Figure S7: Simulation model for the comparison of concentration between flow-through and flow-over a frustule; Figure S8: Simulation model for the flow filed profile in an individual cell and particles adsorption on the surface of the cell; Figure S9: Two-phase flow simulation of water passing through an individual cell of frustule.
