# Supplementary material for: Diatom Frustule Array for Flow-Through Enhancement of Fluorescent Signal in a Microfluidic Chip
Source: Micromachines (Basel). 2021 Aug 26;12(9):1017. doi: 10.3390/mi12091017 (PMC8469004; doi:10.3390/mi12091017)
Supplement: Supplementary file 1 [file micromachines-12-01017-s001.zip › micromachines-1351315-supplementary.pdf]

Supplementary Materials

Diatom frustules array for flow-through enhancement of fluorescent signal in a microfluidic chip

Zhenhu Wang[1], De Gong[1] and Jun Cai[1,*]

[1] School of Mechanical Engineering and Automation, Beihang University, Beijing 100191, China
Correspondence: jun_cai@buaa.edu.cn

1. Fabrication of Si substrate with step-through-holes

The detailed process to etch micro-step-through holes is as follows:

(1) Back cavity etching

The thickness of silicon wafer is 400 μm, which cannot be etched through directly by dry etching technique. Therefore, using photoresist as protective layer, wet etching technique is firstly used to reduce the thickness from 400 μm to 50 μm, at the back side of Si substrate. Besides, a nano-layer of SiO_2/Si_3N_4 was grown on the surface of the wafer, which will be used to etched as the mask to etch the bigger holes.

(2) Etching SiO_2/Si_3N_4 as the mask for the bigger holes

By using photoresist as mask, the SiO_2/Si_3N_4 nanolayer was etched while the Si wafer remained not being etched. Here, the SiO_2/Si_3N_4 nanolayer took over the function of photoresist and would be used as the mask to etch bigger holes.

(3) Etching smaller holes

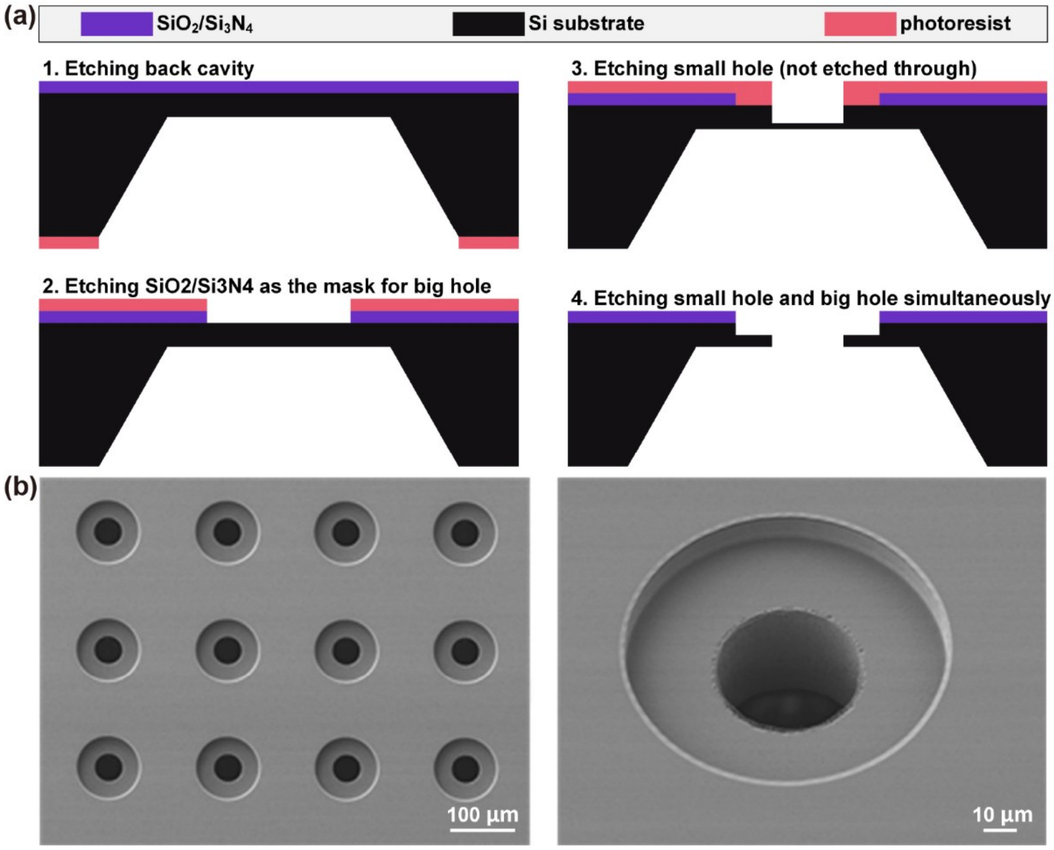

Fig. S1 (a) Procedure to etch micro-step-through holes and (b) SEM image of the holes.

A photoresist layer was re-coating on the wafer and used as the mask to etch smaller holes. But they should not be etched through to avoid the loss of adsorption force to fix Si wafer. In this study, we etched 45 μm in this step.

(4) Etching small holes and big holes simultaneously

The photoresist in step 3 was removed, and the patterned SiO_2/Si_3N_4 nanolayer in step 2 was used as the mask to etch bigger holes to 15 μm depth. In this process, the smaller holes would also be etched to be cut-through. Finally, the SiO_2/Si_3N_4 nanolayer was removed.

2. Coating hot-melt glue in micro holes by under capillary force and bonding frustules

EVA hot-melt glue is insoluble in methylbenzene at room temperature. Firstly, methylbenzene was added into a 50 mL centrifugal tube and heated to 95 °C in a water bath. Then, the EVA powder was added to the methylbenzene at 95°C for 30 min. Finally, the tube was taken out from the water bath and cooling to the room temperature. The concentration of EVA hot-melt glue solution is 0.05 g/mL. This procedure should be finished in a fume hood.

The detailed process of "**spontaneously coating under capillary force**" is described as follows:

① A 1.5×0.7 cm size air-laid paper piece was cut and placed on a slide glass. 40 μL hot-melt glue solution was pipetted on the air-laid paper piece. Next, the Si substrate with step-through holes was placed on the air-laid paper piece carefully.

② After about 3 s, the Si substrate was taken off from the paper and then placed in another clean air-laid paper to absorb the residual solution in the back cavity.

③ Only a small volume of hot-melt glue solution residues on the bottom of the bigger holes.

④ After the methylbenzene volatilizing totally, the hot-melt glue was coated on the bottom of the bigger holes.

⑤ The diatom frustules were manipulated to be placed in the bigger holes and fixed under a negative pressure.

⑥ The electric iron was set to 200 °C and touching the Si substrate for 3 s to heat the hot-melt glue. The negative pressure ensured that the diatom frustules would not detach from the holes.

⑦ After cooling to room temperature, the hot-melt glue bond the frustules with Si substrate.

Before coating hot-melt glue, the Si substrate was treated with a plasma cleaning machine to reduce the contact angle, using air as inlet gas with a 70 W power for 90 s. The pictures of the micro-

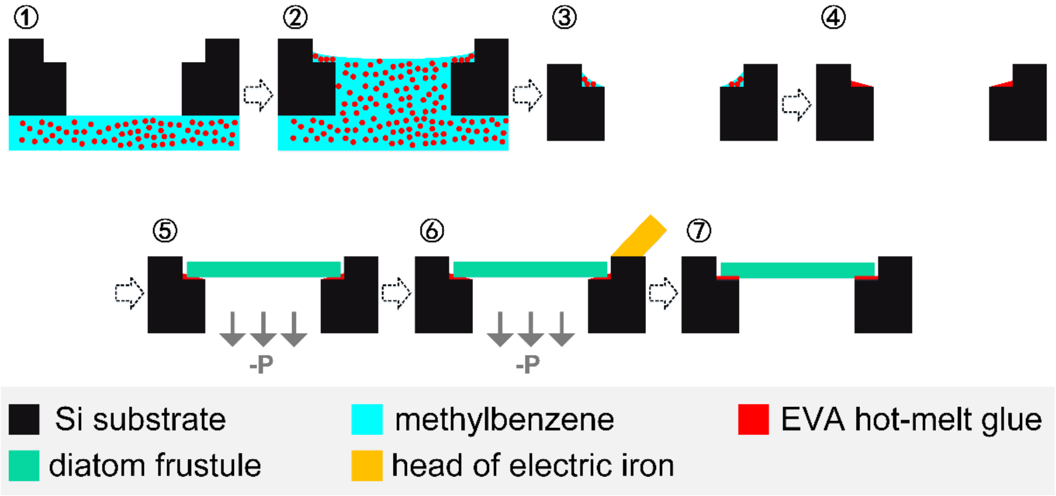

Fig. S2 Coating hot-melt glue in micro holes by under capillary force and bonding frustules

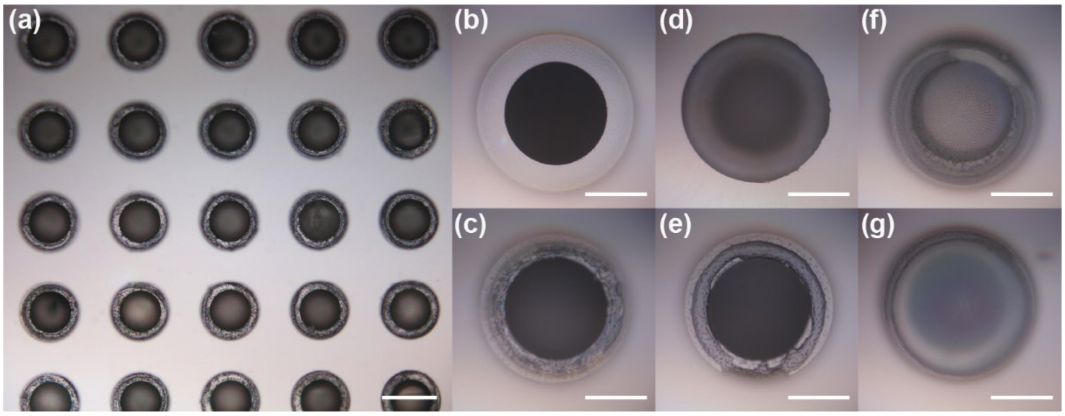

Fig. S3 Optical images of micro holes with hot-melt glue. (a) 5×5 holes array; (b) a clean hole without glue; (c) a micro hole with glue; (d) the surface of Si substrate is clean; (e) a micro hole with glue that has been heated; (f) a concave-up frustules was bonded into the micro hole; (g) a convex-up frustule was bonded to the micro hole. The scale bar is (a) 100 μm and (b)-(g) 50 μm.

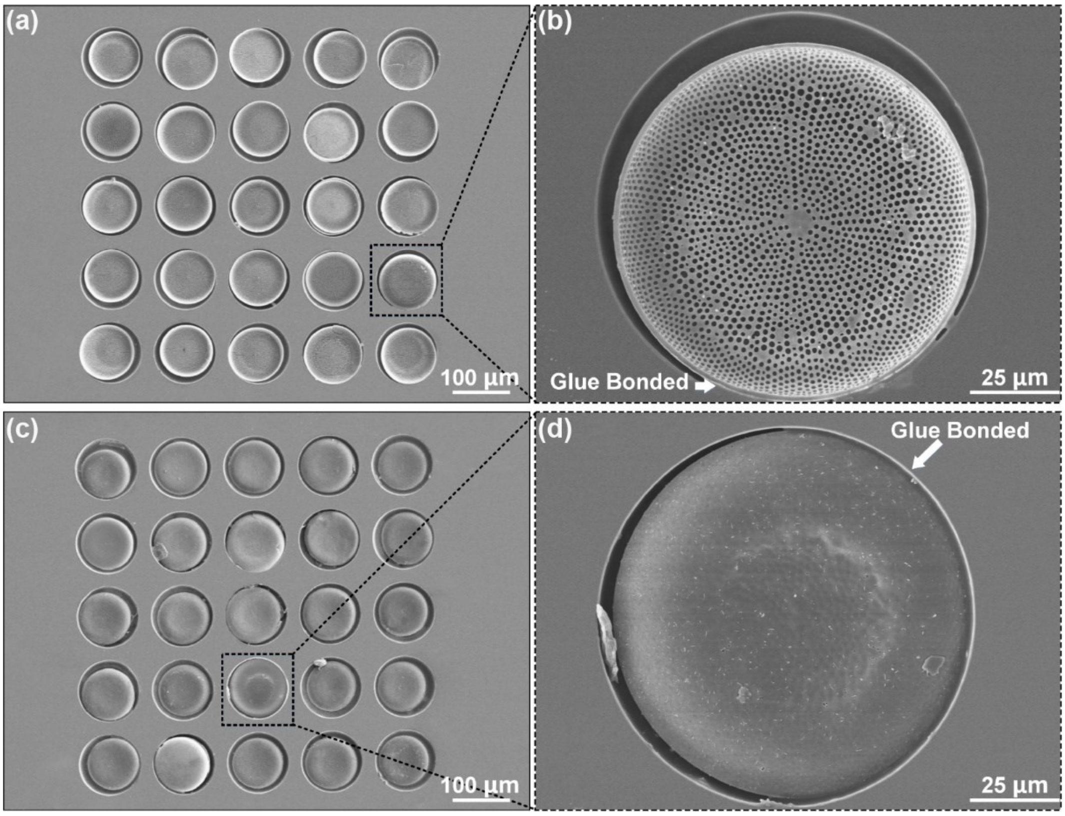

Fig. S4 SEM images of (a) (c) diatom frustules array, that (b) (d) bonded to the Si by hot melt glue.

step-through holes taken by a camera (Canon EOS 500D) mounted on a microscope (Olympus BX53) were shown in Fig. S3. The SEM images were shown in Fig. S4.

3. Patterning frustules by a micromanipulator

The micromanipulator system is shown in Fig. S5. Firstly, the clean diatom frustules were randomly distributed on a clean Si substrate. Next, a capillary glass needle with 30 μm diameter that was mounted on a micromanipulator is operated to approach a single frustule. The frustule would be attracted and attaching to the microneedle. The frustule was moved to the position right above

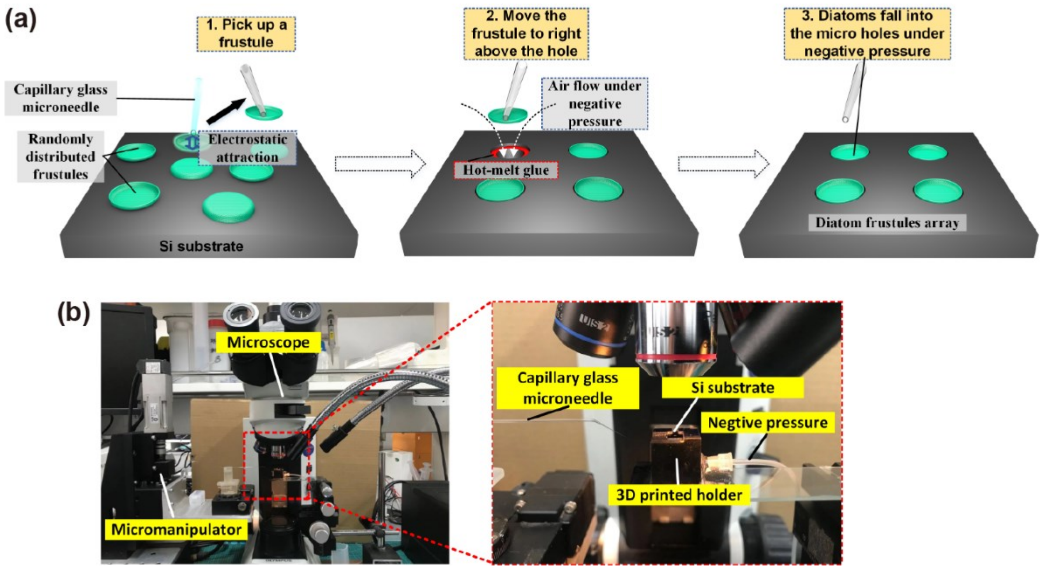

Fig. S5 (a) Schematic illustrations of manipulating diatom frustules into array pattern; (b) Pictures of micromanipulator system.

the micro-step-through holes. Then a negative pressure was exerted from the back of the Si substrate with micro-holes. The frustule would fall into the micro-step-through hole under the negative pressure. Finally, the frustules were bonded to the Si substrate by hot-melt glue as described in "*2.Coating hot-melt glue in micro holes by under capillary force and bonding frustules*". By choosing frustules with different orientation in the first step, a frustules array with different orientation could be obtained.

4. Microfluidic chip bonding

UV curing adhesive was used as the middle layer to bonding four pieces of PMMA and Si substrate. A Portable ultraviolet flashlight was used to cure the adhesive for 90 s, as shown in Fig. S6.

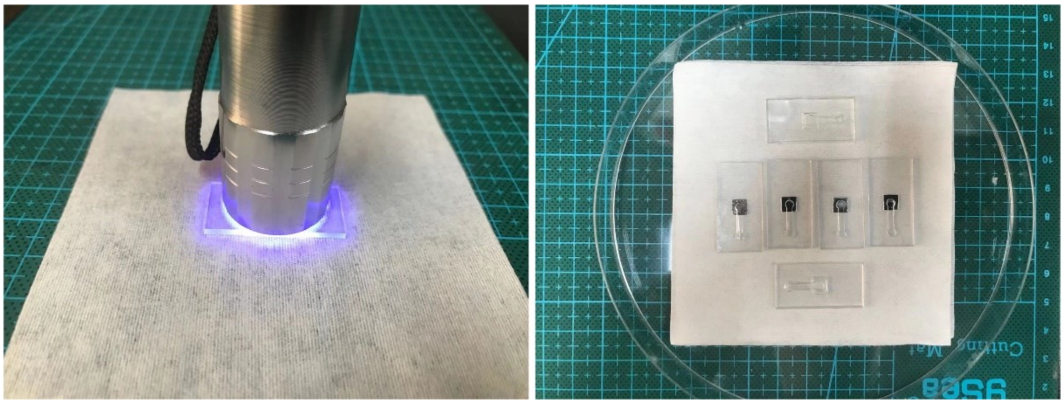

Fig. S6 Microfluidics chip bonding.

5. Simulation model

The simulation model for investigating the distribution of concentration when solution flow through and flow over a frustule is shown in Fig. S7. Laminar Flow (spf), Transport of Diluted Species (tds) and Surface Reaction (sr) module of COMSOL was adopted to conduct the simulation, referring the case "biosensor_design.mph" in the library. In this study, the inlet velocity was set to

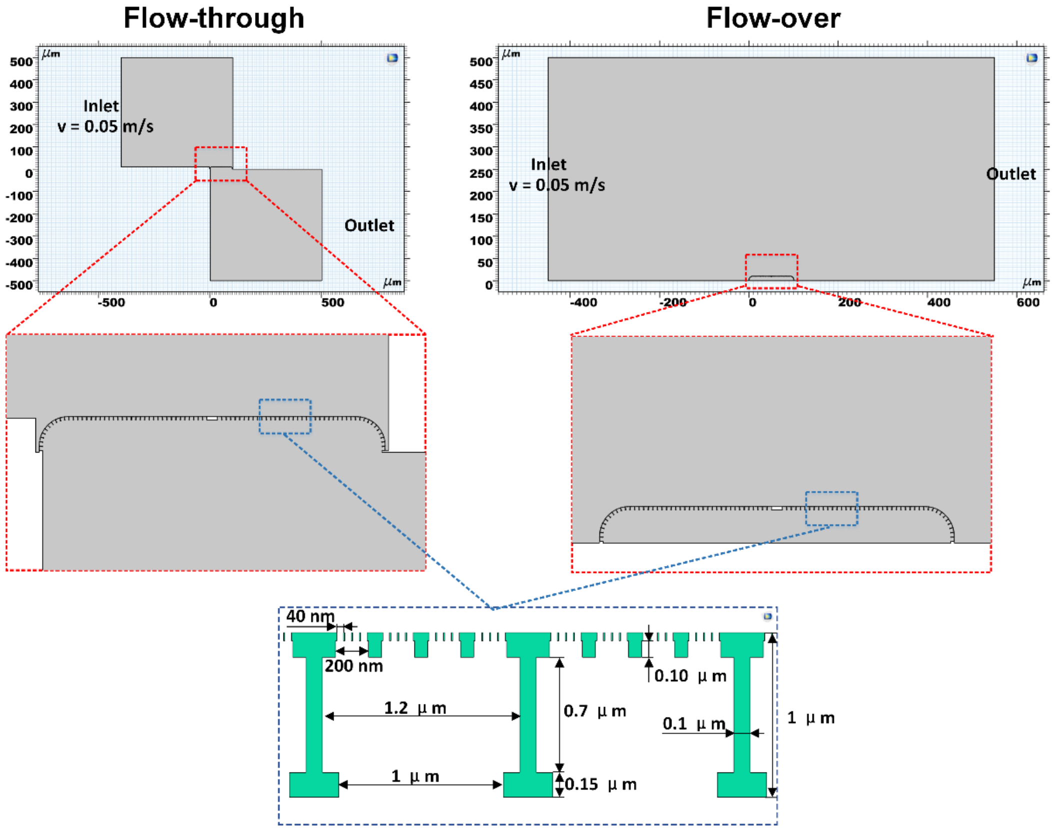

Fig. S7 Simulation model for the comparison of concentration between flow-through and flow-over a frustule

be 0.05 m/s and the inlet concentration was set to be 1 M. For surface reaction module, the in situ density in Surface Properties was set to be 0.1 mol/m^2. A boundary probe was set to out the surface coverage rate of the frustule.

A 3D model was built to investigate the how the nanostructure of the frustules affect the flow filed and adsorption, as shown in Fig. S8, and the results were shown in Fig. 8 and Fig. S9. The yellow arrow represented the interface of water and air. The circle of dot line indicated that for concave-up cell, water would wet the inner side wall thus increased the reaction probability. However, for convex-up cell, a complete interface would regenerate, which would reduce the contact area between solution and inner side wall.

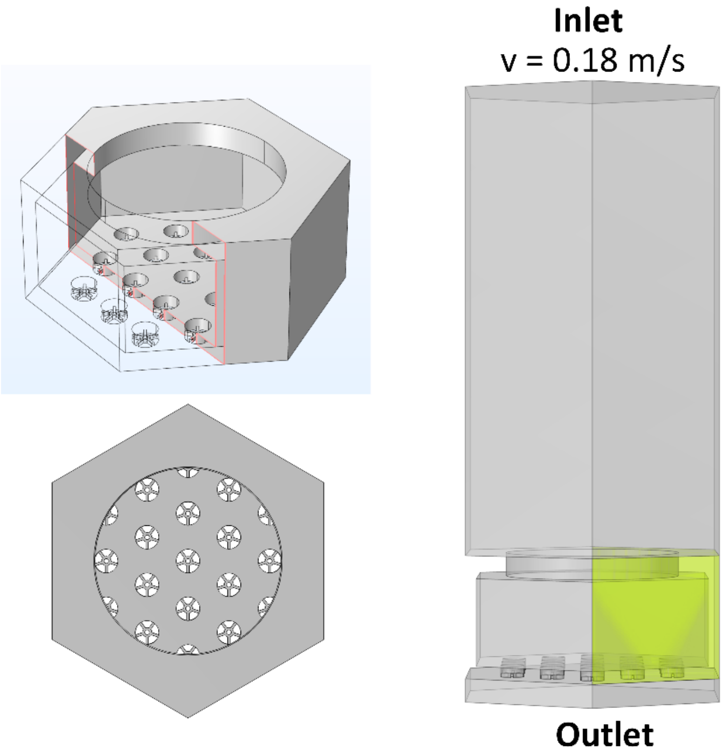

Fig. S8 Simulation model for the flow filed profile in an individual cell and particles adsorption on the surface of the cell

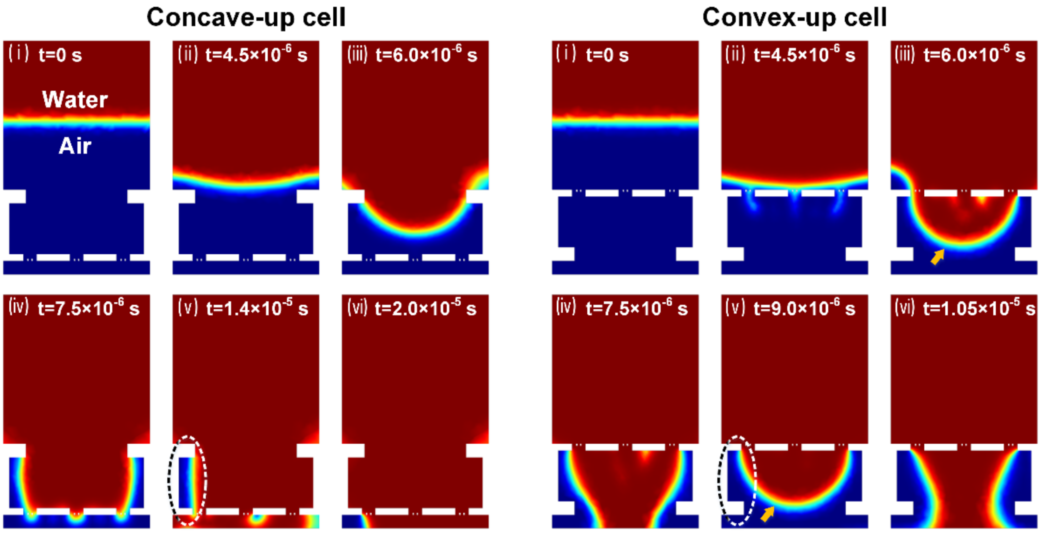

Fig. S9 Two-phase flow simulation of water passing through an individual cell of frustule.
